# Supplementary material for: Plant-Derived Monoterpene Therapies in Parkinson’s Disease Models: Systematic Review and Meta-Analysis
Source: Plants (Basel). 2025 Mar 22;14(7):999. doi: 10.3390/plants14070999 (PMC11990262; doi:10.3390/plants14070999)
Supplement: Supplementary file 1 [file plants-14-00999-s001.zip › Jávega-Cometto et al. Figure supplenmentary legends.pdf]

Figure S1. Forest plot comparing time to fall measurements in the Rotarod behavioral test of parkinsonian animals (PDAs) versus control animals. Effect size is reported as standard mean deviation (SMD), and the variance is reported as the 95% confidence interval (CI). A negative SMD represents less time to fall, whereas a positive SMD represents more time to fall.

Figure S2. Forest plot comparing latency measurements in Catalepsy behavioral test of parkinsonian animals (PDAs) versus control animals. Effect size is reported as standard mean deviation (SMD), and the variance is reported as the 95% confidence interval (CI). A negative SMD represents less time to move, whereas a positive SMD represents more time to move.

Figure S3. Forest plot comparing tyrosine hydroxylase (TH) levels in substantia nigra (SN) and striatum (CPU) of parkinsonian animals (PDAs) versus control animals. Effect size is reported as standard mean deviation (SMD), and the variance is reported as the 95% confidence interval (CI). A negative SMD represents lower TH levels, whereas a positive SMD represents higher TH levels.

Figure S4. Forest plot comparing malondialdehyde (MDA) levels in substantia nigra (SN) and striatum (CPU) of parkinsonian animals (PDAs) versus control animals. Effect size is reported as standard mean deviation (SMD), and the variance is reported as the 95% confidence interval (CI). A negative SMD represents lower MDA levels, whereas a positive SMD represents higher MDA levels.

Figure S5. Forest plot comparing antioxidant enzymes' (superoxide dismutase (SOD) and catalase (CAT)) activity/levels in substantia nigra (SN) of parkinsonian animals (PDAs) versus control animals. Effect size is reported as standard mean deviation (SMD), and the variance is reported as the 95% confidence interval (CI). A negative SMD represents lower levels or activity of antioxidant enzymes, whereas a positive SMD represents higher levels or activity of antioxidant enzymes.

Figure S6. Forest plot comparing cytokines' (interleukin-1 beta (IL-1β), interleukin-6 (IL-6) and tumor necrosis factor (TNF-α)) levels in substantia nigra (SN). of parkinsonian animals (PDAs) versus control animals. Effect size is reported as standard mean deviation (SMD), and the variance is reported as the 95% confidence interval (CI). A negative SMD represents lower cytokine levels, whereas a positive SMD represents higher cytokine levels.
