# Supplementary material for: Plant-Derived Monoterpene Therapies in Parkinson’s Disease Models: Systematic Review and Meta-Analysis
Source: Plants (Basel). 2025 Mar 22;14(7):999. doi: 10.3390/plants14070999 (PMC11990262; doi:10.3390/plants14070999)
Supplement: Supplementary file 1 [file plants-14-00999-s001.zip › Jávega-Cometto et al. Supplementary material. Annex 1.pdf]

Search Strategy and Study Selection Criteria

The following terms were used: ((Parkinson's disease) OR (Parkinson) OR (Parkinsonism)) AND (Ascaridole OR Bornane OR Borneol OR Camphene OR Carene OR Carvacrol OR Carveol OR Carvone OR Citral OR Citronellal OR Citronellol OR Cuminaldehyde OR Ciclocitral OR Cymene OR Eucalyptol OR Fenchol OR Fenchone OR Geranic Acid OR Geraniol OR Geranyl Acetate OR Geranyl pyrophosphate OR Geranylacetone OR Grandisol OR Grapefruit Mercaptan OR Halomon OR Hinokitiol OR Ipsdienol OR Jasmolone OR Lavandulol OR Lavandulyl Acetate OR Limonene OR Linalool OR Linalyl Acetate OR Menthol OR Menthone OR Menthyl Acetate OR Myrcene OR Myrcenol OR Myrtenal OR Myrtenol OR Nerol OR Nerolic Acid OR Ocimene OR 8-oxogeranial OR Perilla Ketone OR Perillaldehyde OR Perillene OR Perillyl Alcohol OR Phellandrene OR Pinane OR Pinene OR Pinocarveol OR Piperitone OR Pulegone OR Rhodinol OR Sabinene OR Safranal OR Terpinen-4-ol OR Terpinene OR Terpineol OR Thujene OR Thujone OR Thymol OR Thymoquinone OR Umbellulone OR Verbenol OR Verbenone OR Wine Lactone). All articles that included these keywords as written from each database were imported into the Zotero software, where duplicates were removed. Screening of the articles was conducted by two independent reviewers, and any discrepancy was resolved by a third party. The exclusion criteria were: 1) Review articles were excluded, 2) Case studies and cross-over studies were excluded, 3) Solely In vitro studies were excluded, 4) Studies done solely on animals different from rats or mice, or human samples were excluded, 5) Studies of experimental models of Parkinson's disease that did not evaluate number/percentage of dopaminergic neurons in relevant structures or motor behavior were excluded, 6) Studies that do not compare control animals with untreated parkinsonian animals were excluded, 7) Studies that did not compare parkinsonian animals without treatment and parkinsonian animals with the use of monoterpenes were excluded, 8) Studies that evaluate therapies with compounds different from monoterpenes were excluded.

Background on PRISMA methodology

The PRISMA methodology, which stands for Preferred Reporting Items for Systematic Reviews and Meta-Analyses, is a set of guidelines designed to improve the quality, transparency, and reproducibility of systematic reviews and meta-analyses, particularly in the healthcare and medical sciences. The main components of PRISMA are a checklist that provides recommendations for the reporting of systematic reviews, and a flow diagram which represents the study identification and selection process. PRISMA aims to ensure that systematic reviews and meta-analyses are conducted and reported in a transparent and reproducible manner, enhancing the credibility of research findings [1].

The detailed procedures, including study selection criteria and data extraction methods are outlined in the registered protocol available in the International Prospective Register of Systematic Reviews (PROSPERO) database (CRD42024592555).

References

[1] Page, M. J., McKenzie, J. E., Bossuyt, P. M., Boutron, I., Hoffmann, T. C., Mulrow, C. D., ... & Moher, D. (2021). The PRISMA 2020 statement: an updated guideline for reporting systematic reviews. bmj, 372.
