# Supplementary figures and images for: Plant-Derived Monoterpene Therapies in Parkinson’s Disease Models: Systematic Review and Meta-Analysis
Source: Plants (Basel). 2025 Mar 22;14(7):999. doi: 10.3390/plants14070999 (PMC11990262; doi:10.3390/plants14070999)

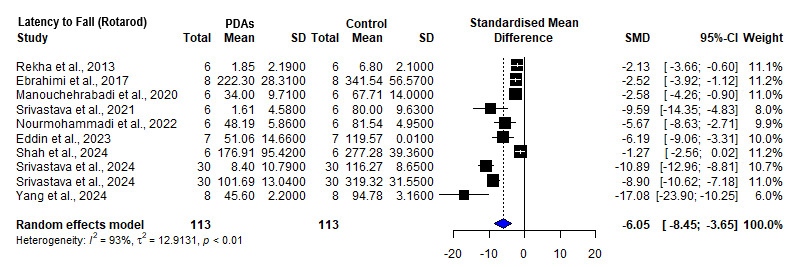

Supplement: Supplementary file 1 [file plants-14-00999-s001.zip › Jávega-Cometto et al, Figure S1.jpg]

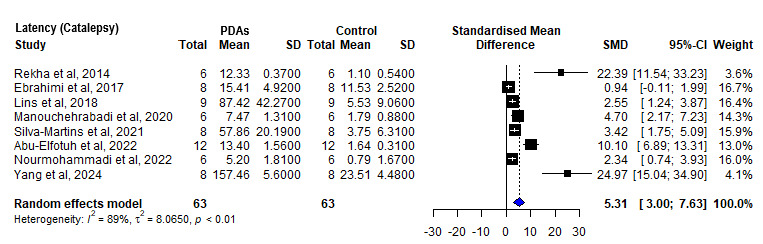

Supplement: Supplementary file 1 [file plants-14-00999-s001.zip › Jávega-Cometto et al, Figure S2.jpg]

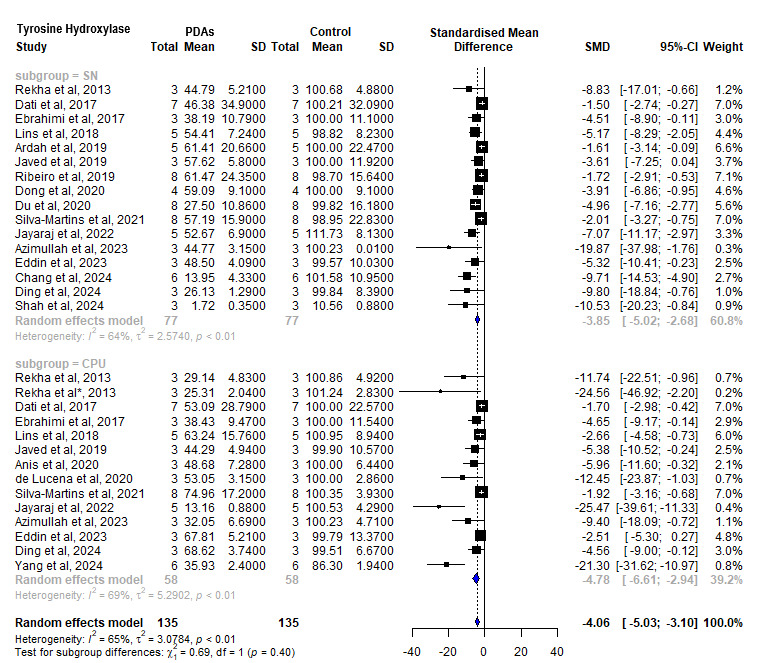

Supplement: Supplementary file 1 [file plants-14-00999-s001.zip › Jávega-Cometto et al, Figure S3.jpg]

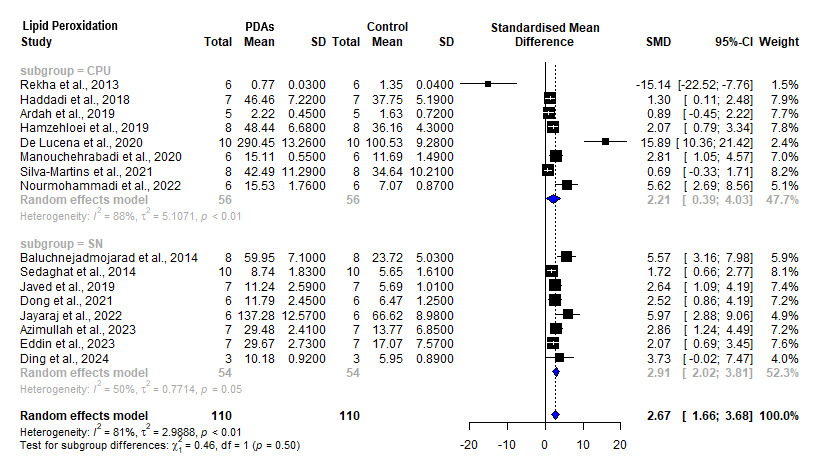

Supplement: Supplementary file 1 [file plants-14-00999-s001.zip › Jávega-Cometto et al, Figure S4.jpg]

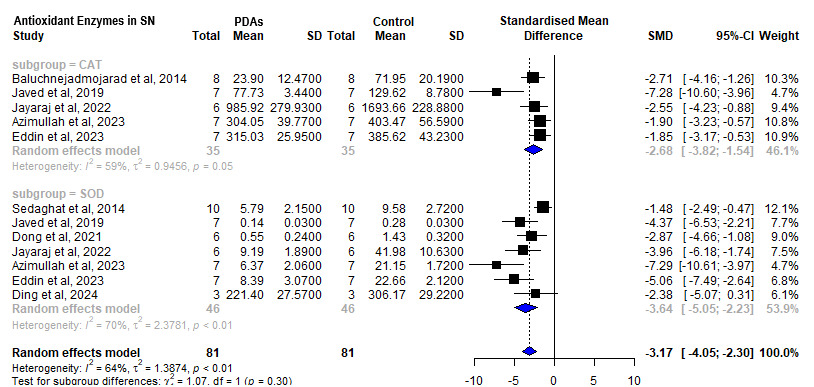

Supplement: Supplementary file 1 [file plants-14-00999-s001.zip › Jávega-Cometto et al, Figure S5.jpg]

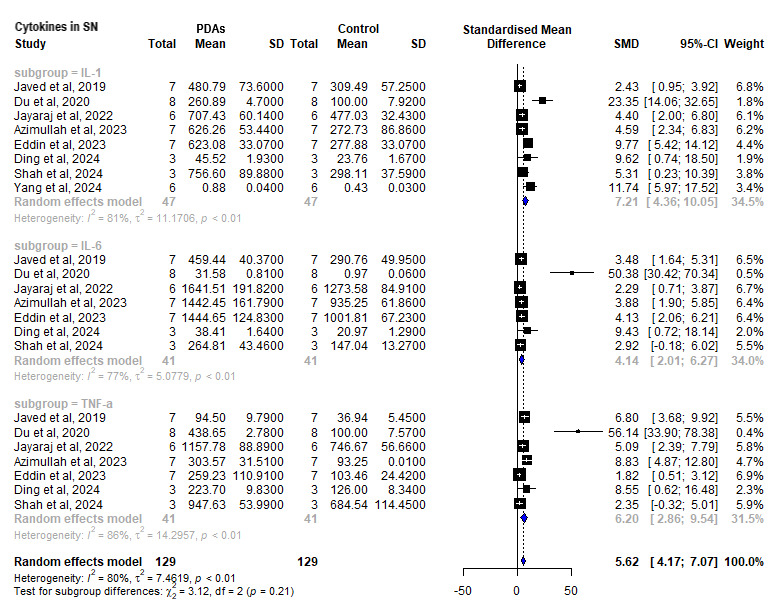

Supplement: Supplementary file 1 [file plants-14-00999-s001.zip › Jávega-Cometto et al, Figure S6.jpg]
